# Early-Onset Breast, Cervical, Colorectal and Stomach Cancer Across the States of India, 1990–2023: Divergent Trends and a Mismatch Between Burden Rate and Caseload

**DOI:** 10.3390/cancers18213580

**Published:** 2026-11-07

**Authors:** Rajesh Kamath, Sanjay B. Kini, Vidya Prabhu, Andria Sirur

**Affiliations:** 1 Department of Healthcare and Hospital Management, Prasanna School of Public Health, Manipal Academy of Higher Education, Manipal 576104, India; rajesh.kamath@manipal.edu; 2 Department of Community Medicine, Kasturba Medical College, Manipal Academy of Higher Education, Manipal 576104, India; sanjay.kb@manipal.edu; 3 Department of Global Public Health Policy and Governance, Prasanna School of Public Health, Manipal Academy of Higher Education, Manipal 576104, India; 4 Manipal School of Commerce and Economics, Manipal Academy of Higher Education, Manipal 576104, India

**Keywords:** early-onset cancer, breast cancer, cervical cancer, colorectal cancer, stomach cancer, India, states, Global Burden of Disease, Socio-Demographic Index, health inequality

## Abstract

Background/Objectives: Cancer diagnosed before the age of 50 is rising across many countries, and the rise is not accounted for by population growth alone. India carries one of the world’s largest cancer burdens, and it varies greatly between its states, variation camouflaged by the national average. We characterised early-onset breast, cervical, colorectal and stomach cancer across the states of India. Methods: Secondary ecological analysis of Global Burden of Disease Study 2023 estimates, ages 15–49 years, 1990–2023; rates per 100,000 population of both sexes. Trends were summarised as the estimated annual percentage change, with autocorrelation-robust confidence intervals, and by segmented regression. Gradients across 30 states were assessed against the 2023 Socio-Demographic Index (SDI). Case growth was decomposed into population growth, within-band ageing and rate change. Results: In 2023, breast cancer had the highest early-onset incidence, 9.63 per 100,000 (19.63 per 100,000 women), and cervical cancer 8.18 (16.87). Breast incidence rose 2.72% per year and colorectal 1.11%; stomach fell 0.52%. Cervical incidence fell until 2011 and rose unevenly thereafter, with a near-zero net trend (−0.09% per year). Within-band standardisation shifted it downward (−0.39%; 95% confidence interval −0.87 to 0.09), and separating within-band ageing turned its rate-change share negative (−8.1%). Breast cases rose from 15,550 to 76,806: 59.0% of the rise was rate change, 51.8% after separating ageing. Point estimates suggested a stronger inverse SDI gradient for cervical disability-adjusted life years (ρ = −0.43) than incidence (ρ = −0.31). Neither survived multiple-testing correction, and the two were not distinguishable. No association was detected between burden rate and caseload across the states (ρ = +0.02). Conclusions: The early-onset cancer burden in India is not uniform. Breast cancer cases are growing faster than the population, with a rising recorded rate. The cervical gradient is exploratory. The states with the highest rates and the states with the most cases differ, and each answers a different planning question.

## Simple Summary

Cancer is being diagnosed more often before the age of 50, in the years when people are working and raising families. India has one of the world’s largest populations of young adults but usually appears in international studies as a single national figure. We used global estimates for 1990 to 2023 to describe cancers of the breast, cervix, colon and rectum, and stomach for each Indian state in adults aged 15 to 49 years. The four are moving in different directions. Breast cancer cases have grown faster than the population because the recorded rate has risen, through more disease, better detection, or both. Cervical cancer fell for two decades and has risen, unevenly, since. Its burden appeared heavier in states lower on a development index, but that pattern needs confirmation. The states with the highest rates are not the states with the most patients, and planning needs both.

## 1. Introduction

The incidence of cancer diagnosed before the age of 50, early-onset cancer, has risen across many countries over the past three decades, and part of that rise appears genuine rather than an artefact of wider screening [1]. Across 204 countries, the age-specific incidence of early-onset cancer rose between 1990 and 2021 while its death rate fell [2]. Where the rate itself is climbing, something beyond demography is at work. Either the underlying risk is rising or the disease is being found earlier. Observational data rarely separate the two. Either way, it is a shift in who is diagnosed, and when. It moves a share of the cancer burden from the old to the young, into the years when people work, earn, and raise children.

The concern is sharpest for a small group of cancers. Breast, colorectal, and stomach cancer are among the malignancies that shape the cancer burden of Asia [3], and breast and cervical cancer are the two leading cancers among Indian women [4]. At all ages, the four together caused an estimated 216,310 of India’s 901,828 cancer deaths in 2024, about a quarter: breast cancer 96,100, stomach cancer 45,392, colorectal cancer 38,499, and cervical cancer 36,319 [5]. Colorectal cancer is the cancer driving global alarm about early onset, rising in country after country among adults in their thirties and forties [6,7]. Stomach cancer, by contrast, is a cancer of an earlier epidemiological era: among adults aged 15–49 its incidence has fallen worldwide over three decades, faster than that of any other gastrointestinal cancer [8]. Grouped together, these four cancers describe the early-onset transition in a developing country: two cancers of women, one cancer moving with affluence, and one moving against it. Lung cancer was not included. It causes more cancer deaths in India than any other site [5], but it is predominantly a cancer of older adults and of long tobacco exposure. Younger patients, variously defined, make up 1–10% of non-small cell lung cancer patients in Asian populations [9], and lung cancer is not among the cancers whose early-onset incidence has been reported to be rising [1].

India is central to this story and largely absent from its evidence. About 798 million Indians are aged 15–49 on the estimates analysed here [10]. That is a population comparable to the whole of Europe. It is this group that an earlier-onset cancer burden falls on. India carries one of the world’s largest cancer burdens, and the burden and its causes vary greatly between the states [11]. Many states have populations the size of large countries. They sit at markedly different stages of the epidemiological transition: for five of the ten leading causes of disease burden, the highest state-specific rate is at least five times the lowest [12]. The quality of public primary health care differs sharply across states, and it is lowest and most variable in the states the government classifies as low-performing [13]. Yet in the multi-country cancer literature, India appears as a single national unit: one line on a cross-country chart [1,3]. Variation of this magnitude is camouflaged by the national average.

The camouflage matters, because the drivers of these four cancers are distributed unevenly across the country. About 70% of India’s cancer burden is attributed to potentially modifiable and preventable risk factors, and cervical cancer, where a single infection accounts for most cases and both a vaccine and a screening test exist, is the clearest case [14]. Screening and human papillomavirus (HPV) vaccination determine much of what happens to cervical cancer. Both are available in India, and the evidence for HPV vaccination from Indian cohorts is strong [15], but their delivery has been limited and uneven [16]. High body mass index and high fasting plasma glucose have risen in importance among the risk factors for early-onset colorectal cancer over three decades [17]. The reproductive and metabolic profile they belong to is later childbearing, lower parity and higher body mass index. It varies markedly across the states of India [18]. Stomach cancer is tied to Helicobacter pylori infection and to diet, both of which change with development [8]. If four cancers are driven by different forces, and those forces are spread unevenly across the country, then the early-onset burden is unlikely to be uniform. It can be expected to vary by state, and a national analysis cannot show how.

Financial protection is uneven too, and that is the second reason the sub-national view matters. Cancer carries the highest adjusted odds of distress financing of any illness group in India and imposes a substantial economic burden on households [19,20]. Catastrophic health expenditure varied eight-fold across states in 2014 [21]. Among poor households, out-of-pocket expenditure per hospitalisation episode in public facilities was at least twice as high in the poorer states as in Tamil Nadu in 2014 [22]. Neither the Rashtriya Swasthya Bima Yojana nor Ayushman Bharat covers outpatient care [23,24], and cancer expenditure outside a covered treatment package is not reimbursed. This analysis measures burden and not expenditure, and no claim about financial protection is tested here. It is why the question is worth asking geographically: if burden and weak protection concentrate in the same states, the household cost compounds.

Locating where the early-onset burden is heaviest is therefore a prior step to asking where prevention and financial protection are most needed. Global Burden of Disease (GBD) analyses have mapped cancer across India’s states for all ages [11]. A nationally representative mortality survey has described the social distribution of cancer deaths across the states [25], and a national assessment has described the growing burden and its social context [26]. One GBD analysis has traced cervical cancer incidence and mortality across the states at all ages [27], and registry-based estimates place the heaviest cervical burden in the northeastern states [28]. Early-onset cancer has been characterised globally [1,2]. For colorectal cancer, a state-level GBD analysis of India has reported early-onset incidence at ages 15–49 alongside its all-age estimates [29]. To our knowledge, and on a PubMed search updated in October 2026, no analysis has characterised early-onset breast, cervical, colorectal, and stomach cancer together across the states of India, and none has asked whether early-onset occurrence and early-onset mortality follow the same socio-demographic gradient. This study adds three things: a state-level characterisation of the four cancers at ages 15–49 on one consistent set of estimates; a direct test of whether their incidence and mortality share a socio-demographic gradient; and a comparison of burden rate with absolute caseload, the two quantities that state planning needs. The gap has a practical cost. The decisions that follow from this burden are taken state by state and not nationally: where to sequence human papillomavirus vaccination and cervical screening, where to place early breast cancer detection capacity, and which states to prioritise when financial protection is extended. Those decisions are currently taken against a national average that fits no state well.

Early-onset cancer is defined here as malignancy in adults aged 15–49, the operational age band of GBD-based early-onset analyses [2,8]. Using GBD 2023, we characterise breast, cervical, colorectal, and stomach cancer across the states of India from 1990 to 2023. We quantify the trend in each and describe its shape. We decompose the change in cases into population growth, within-band ageing, and change in the observed rate. We describe the socio-demographic gradient across states separately for incidence, deaths, and disability-adjusted life years (DALYs). The incidence and mortality gradients are compared directly rather than inferred from each coefficient’s significance. The aim is to replace one national number with a map: where each cancer is rising, where it is falling, and where it falls hardest.

## 2. Materials and Methods

### 2.1. Study Design and Data Source

This is a secondary ecological analysis of published, aggregated modelled estimates. It combines longitudinal trend analyses for 1990–2023 with a cross-sectional ecological analysis of the states in 2023. Estimates were extracted from the Global Burden of Disease Study 2023 Results tool on 15 July 2026 [10]. GBD 2023 is the most recent completed round of the study and its estimates end in 2023, so no later year is available on a consistent basis. The estimation methods are described in the GBD 2023 systematic analyses of disease and injury burden and of cancer burden [30,31]. The Socio-Demographic Index (SDI) for 2023 was obtained from the Global Health Data Exchange and matched to the burden estimates by GBD location name. The GBD location identifiers are listed in File S1. Both sources are open access and were used without modification. Reporting follows the Guidelines for Accurate and Transparent Health Estimates Reporting (GATHER), the instrument developed for studies based on modelled health estimates [32]. The completed checklist is provided as File S3. The study has no individual participants and fits no new estimation model. The participant-level items of the Strengthening the Reporting of Observational Studies in Epidemiology (STROBE) checklist [33] therefore do not apply: setting, participants, bias, study size, and quantitative variables are all defined at the level of individuals. The full query specification is recorded in the README sheet of File S1.

### 2.2. Case Definition and Variables

**Figure 1:**
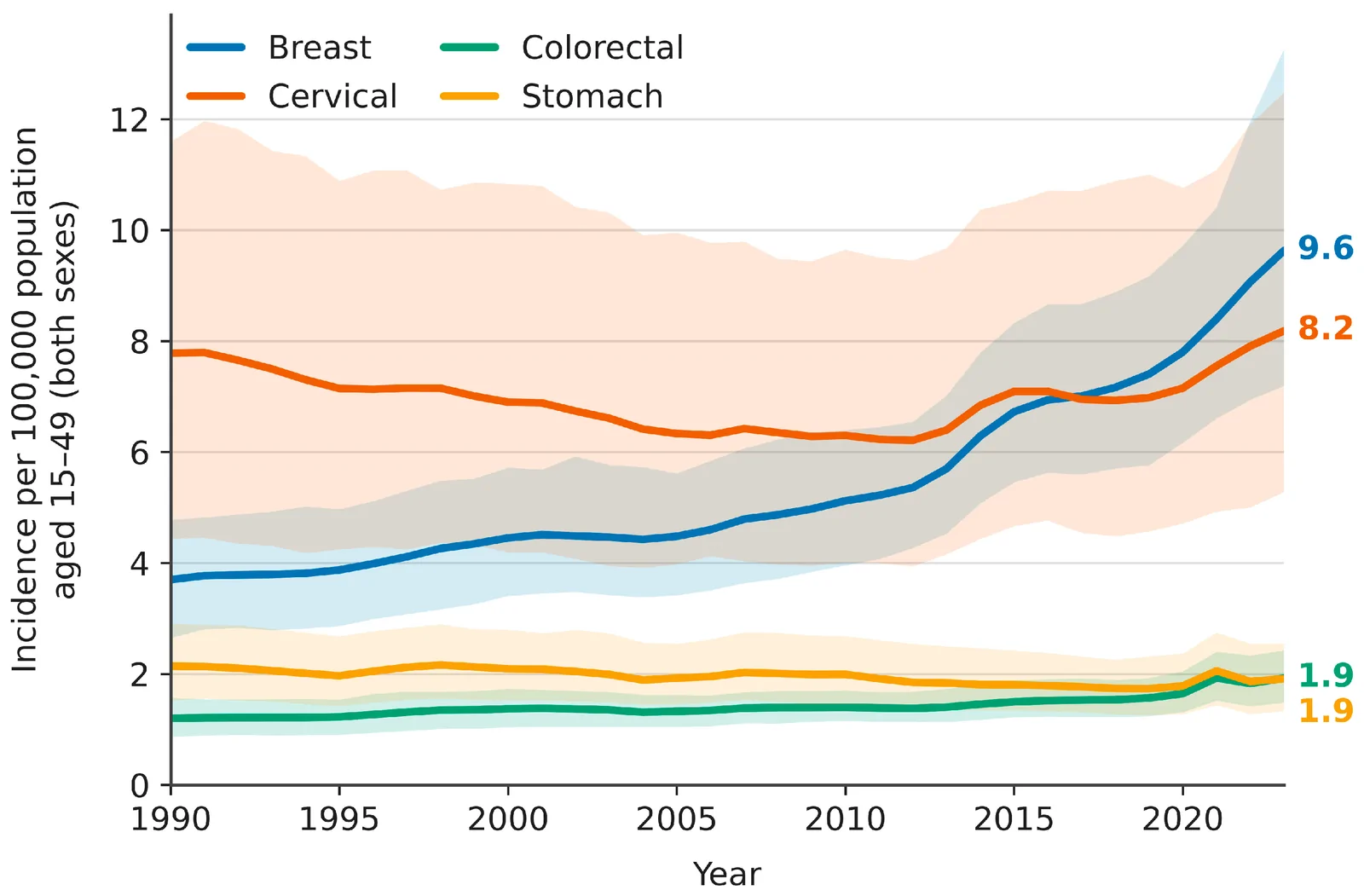
Early-onset (ages 15–49) cancer incidence in India, 1990–2023: incidence per 100,000 population aged 15–49, both sexes, four cancers. Lines are GBD 2023 point estimates and bands are GBD 95% uncertainty intervals. Source: GBD 2023 [10].

Early-onset cancer was defined as malignancy in adults aged 15–49 years [2,8]. Four cancers were studied: breast, cervical, colon and rectum (reported here as colorectal), and stomach cancer. For each, we extracted incidence, deaths, and DALYs, in count and age-specific rate metrics, for both sexes combined and by sex, for every year from 1990 to 2023. Rates are per 100,000. Breast and cervical cancer cases arise almost entirely in women, but every rate in the main analysis uses the total population aged 15–49, of both sexes, as the denominator, so that the four cancers are directly comparable. Breast and cervical rates are therefore about half the conventional female-specific rates and should not be compared numerically with published female rates. Female-specific rates are reported alongside in Table 1 and Table S6, which also repeat each headline result on the female denominator. Rates, not counts, are used to compare states, because a rate divides each state’s cases by its own population and so removes differences in population size; counts are used where the question is the absolute number of patients. Incidence at ages 15–49 approximates diagnosis before the age of 50. Deaths and DALYs at ages 15–49 are those occurring within the band. They do not represent the lifetime burden of patients diagnosed before 50, part of which falls at older ages. Each estimate carries a 95% uncertainty interval (UI), retained for every observation in the supplementary extract and shown as bands in Figure 1.

### 2.3. Locations

Estimates were extracted for India and for its GBD subnational units. The state-level analyses, the state trends, the socio-demographic gradient and the composite burden ranking use the 30 named states and union territories. The residual “Other Union Territories” aggregate is not a geographical unit with an interpretable Socio-Demographic Index and is excluded from all three. It is retained in the supplementary extract. No urban or rural splits were used.

### 2.4. Statistical Analysis

Trends in each rate series were summarised as the estimated annual percentage change (EAPC). A log-linear model, ln(rate) = α + β·year + ε, was fitted to the 34 annual observations for each cancer and location, and the EAPC computed as 100 × (exp(β) − 1) [34]. The EAPC is an average trend across the whole period and does not test for changes in slope. Residuals of the annual series were strongly autocorrelated (national series: lag-1 correlation 0.58 to 0.95, Durbin–Watson statistic 0.10 to 0.83; Table S1). The 95% confidence intervals (CIs) were therefore computed from Newey–West heteroskedasticity- and autocorrelation-consistent standard errors with a bandwidth of three years and a small-sample correction, using the t distribution with 32 degrees of freedom [35]. Intervals assuming independent errors, and Newey–West intervals at bandwidths of six and ten years, are given in Table S1 for comparison. Goodness of fit was summarised by R^2^, and departure from a single log-linear trend was tested by adding a quadratic term in year, with Newey–West standard errors of the same specification. Because a single trend did not describe most national series adequately, each national series was also fitted by segmented log-linear regression of the Joinpoint type [36], with up to three joinpoints placed at least three years from either end and from each other and their number chosen by the Bayesian information criterion. Segments are summarised by their annual percentage change and the full period by the average annual percentage change (AAPC), with Newey–West CIs conditional on the selected joinpoints (Table S8). State-level EAPCs are assessed against their own 95% CIs, and Benjamini–Hochberg q-values across all 120 state-by-cancer estimates, computed as described below, are reported in Table S2.

Association between state burden in 2023 and the state Socio-Demographic Index was assessed by Spearman rank correlation, on the unrounded SDI values of the GBD 2023 release, which are provided in File S1. The health concentration index, C = 2·cov(y, R)/μ, where R is the fractional rank of the state by SDI and μ is the mean of the burden measure, was computed alongside it [37]. A negative concentration index indicates burden concentrated among the lower-SDI states. In the primary index, every state counts equally, so it describes inequality between states. A population-weighted index, in which R is the population-weighted fractional rank, describes inequality between people and is reported in Table S3.

The 1990–2023 change in incident cases was decomposed into a population-growth component and a rate component by a Das Gupta–type factorisation [38], with the implied population obtained as the ratio of the count to the rate. The rate component measures change in the observed rate. It does not distinguish change in underlying risk from change in detection, registration, or modelling. Rate and caseload were compared by ranking the states on the sum of the four cancer-specific DALY rates in 2023 and on their summed incident case count. The summed DALY rate is a descriptive summary measure constructed for this analysis. It is not a validated index of cancer-control need: the four cancers differ in sex distribution, aetiology, prevention, prognosis and treatment; a state can rank high through a single cancer, and cancer-specific results remain the basis for disease-specific planning. The comparison was repeated on matched measures, incidence rate against incident count and DALY rate against DALY count, for the four cancers combined and for each separately.

Because the interpretation of the cervical result rests on a contrast between the incidence gradient and the mortality gradient, the two were compared directly by a paired bootstrap: the 30 states were resampled with replacement 10,000 times, both Spearman coefficients were recomputed in each replicate, and the distribution of their difference summarised as a 95% percentile interval and a two-sided *p*-value. Two-sided *p* < 0.05 was taken as nominally significant. Twelve gradient tests were performed: four cancers by three measures. Because no single test was pre-specified, the Benjamini–Hochberg false discovery rate [39] was applied across all twelve, and adjusted q-values are reported alongside the unadjusted *p*-values in Table S3. With 30 states, power for the ecological gradient analyses is limited. At 80% power and two-sided α = 0.05, the smallest detectable coefficient is |ρ| = 0.49, so non-significant directional associations are reported as such rather than as evidence of no association. The SDI analysis is exploratory.

There is no public release of GBD posterior draws through the Results tool, so the uncertainty intervals could not be propagated exactly. As an approximation, each published 2023 estimate, and each 1990 and 2023 national estimate used in the two-factor decomposition, was represented by a log-normal distribution centred on its point estimate, with a standard deviation derived from its UI, and 2000 independent draws were used to recompute the state ranking, the cervical SDI gradients, the rate–caseload correlations, and the two-factor decomposition (Table S9). The three-factor decomposition was not simulated. The trend CIs are conditional on the GBD point estimates and do not include the uncertainty of the underlying estimates. Because the 15–49 band is wide and its internal age composition changed over the period, a sensitivity analysis was performed on a second extract of the same GBD 2023 release, taken for India by seven five-year age groups (15–19 to 45–49 years) on 6 September 2026. The five-year counts reconcile exactly with the aggregate band. Rates were directly standardised within the band, to the World Health Organization (WHO) World Standard Population truncated to those seven groups [40] and, as an alternative, to India’s own 1990 within-band composition, and both are reported. The change in incident cases was re-decomposed by a three-factor Das Gupta factorisation into population size, within-band age structure, and age-specific rates. Results are given in Table S7. Analyses used Python 3.12 (pandas, numpy, scipy, statsmodels, matplotlib, openpyxl). The analysis-ready extract is provided as File S1 and the complete analysis script as File S2.

## 3. Results

### 3.1. Early-Onset Cancer Burden in India, 2023

In 2023, breast cancer had the highest early-onset incidence in India, at 9.63 per 100,000 population aged 15–49 (95% UI 7.19 to 13.26; Table 1), or 19.63 per 100,000 women (14.69 to 27.19). Cervical cancer followed at 8.18 (5.28 to 12.48), or 16.87 per 100,000 women (10.88 to 25.72). The uncertainty intervals of the two overlap widely, so the ordering of breast above cervical rests on the point estimates. Colorectal and stomach cancer were far less common, at 1.92 and 1.91 per 100,000. Death rates per 100,000 were 3.46 for breast cancer (2.55 to 4.56), 2.50 for cervical (1.67 to 3.69), 1.51 for stomach (1.06 to 2.02), and 1.18 for colorectal cancer (0.91 to 1.49). Ranked by DALYs, the order changes at the bottom: breast 178.2, cervical 127.5, stomach 74.4, and colorectal 59.5 per 100,000. Stomach cancer carries more deaths and more health loss than colorectal cancer at almost the same incidence. Within this age band that is consistent with a higher case fatality, but these estimates do not establish it. Taken together, the four cancers accounted for 172,673 incident cases, 68,994 deaths and 3.51 million DALYs among Indians aged 15–49 in 2023, against 62,301 incident cases in 1990.

EAPC estimated annual percentage change, CI confidence interval, DALYs disability-adjusted life years, SDI Socio-Demographic Index, UI uncertainty interval. Source: GBD 2023. Rates are per 100,000 population aged 15–49, both sexes combined, except the female-specific row, which is per 100,000 women aged 15–49. UIs are GBD 95% uncertainty intervals. CIs are 95% confidence intervals from the secondary analysis, computed with Newey–West autocorrelation-consistent standard errors and conditional on the GBD point estimates. The within-band standardised EAPC is directly standardised across the seven five-year age groups of the 15–49 band (Table S7). The rate-change share is the rate component’s share of the net change in incident cases. In the two-factor decomposition, the population-growth component makes up the remainder, and in the three-factor decomposition, population size and within-band age structure do. A negative rate-change share means the observed rate fell while the case count rose through demographic change alone. *p*, unadjusted; q, Benjamini–Hochberg adjusted across the twelve gradient tests (four cancers by three measures). None of the twelve survives correction: the cervical deaths and DALY coefficients are nominally significant only. All twelve coefficients are given in Table S3.

### 3.2. Divergent National Trends, 1990–2023

The four trends diverged (Figure 1). Breast cancer incidence rose by 2.72% per year (95% CI 2.19 to 3.25) and colorectal cancer by 1.11% (0.75 to 1.48). Stomach cancer fell by 0.52% (−0.75 to −0.28). Cervical cancer showed no material net trend: −0.09% per year (−0.61 to 0.43), an interval that includes zero. These intervals allow for the strong autocorrelation of the annual series. Intervals assuming independent errors were narrower and are given, with the national trends in deaths and DALYs, in Table S1. A single log-linear trend did not describe the full period well for breast or cervical cancer (quadratic term *p* < 0.001 for each, with Newey–West standard errors; Table S1). For colorectal cancer, the evidence of departure was weaker (*p* = 0.018). The segmented fits show the shape (Table S8). Breast incidence rose by 1.64% per year to 2012 and faster thereafter. Cervical incidence fell by 1.15% per year from 1990 to 2011 and has risen since in two steep steps, in 2011–2015 and 2019–2023, separated by a small decline in 2015–2019: the near-zero EAPC is the net of a two-decade decline and a later, uneven rise. The average annual percentage change from the segmented fit is also small, at +0.18% per year, though opposite in sign. Its interval is conditional on the selected joinpoints and so understates the uncertainty (Table S8). Colorectal incidence accelerated after 2018, to 5.20% per year. Stomach incidence fell until 2018 and turned upward in the last five years. Short, steep segments at the end of a modelled series should be read with caution, because they can reflect changes in the input data rather than in disease. Over three decades, breast cancer incidence went from 3.7 to 9.6 per 100,000, while cervical cancer went from 7.8 to 8.2. Colorectal cancer rose from 1.20 to 1.92 per 100,000 over the same period. Stomach cancer fell from 2.14 to 1.91. On the point estimates, colorectal and stomach cancer exchange places over the period, though their 2023 uncertainty intervals overlap almost entirely and the ordering between them in 2023 should not be read as established. Two cancers of women, in the same country and the same age band, followed different paths.

### 3.3. Population Growth or Rate Change

**Figure 2:**
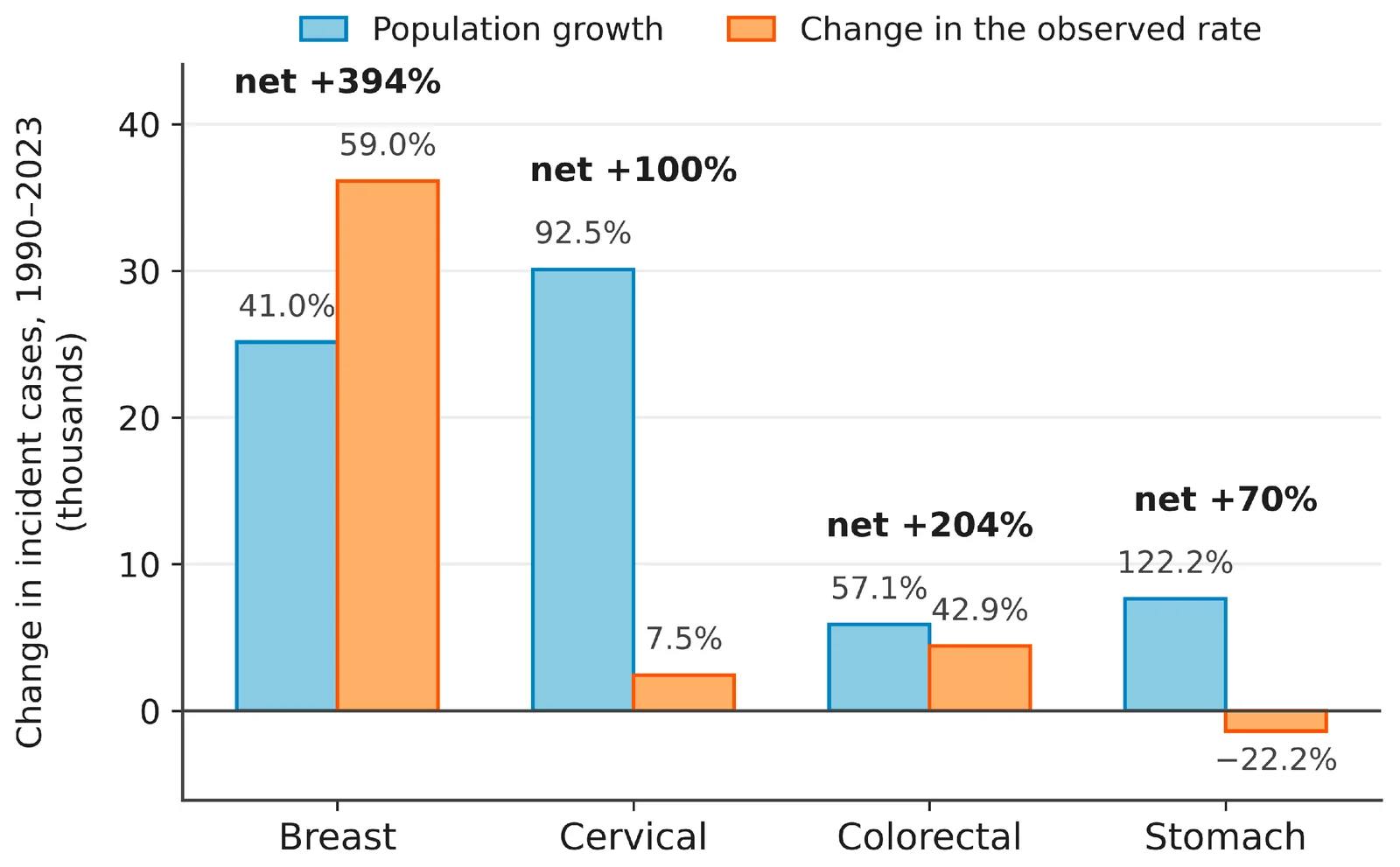
Drivers of the change in early-onset (ages 15–49) incident cancer cases in India, 1990–2023: population-growth and rate components of the two-factor decomposition, by cancer, both sexes. Bars show each component in thousands of cases, plotted as signed contributions that sum to the net change in cases. The label on each bar is that component’s share of the net change, and the label above each pair is the percentage change in cases. For stomach cancer, the rate component is negative: the case count rose through population growth alone while the observed rate fell. The rate component measures change in the observed rate and does not distinguish change in underlying risk from change in detection, registration, or modelling. Source: GBD 2023 [10].

Incident breast cancer cases rose from 15,550 in 1990 to 76,806 in 2023, an increase of 394%. Of that increase, 41.0% is attributable to population growth and 59.0% to change in the observed rate (Figure 2). The picture for cervical cancer is the opposite: cases doubled, from 32,715 to 65,273, but 92.5% of that rise is population growth and only 7.5% is rate change. Colorectal cancer cases rose 204%, of which 42.9% is rate change. Stomach cancer cases rose 70% while the rate fell, so the rate component is negative at −22.2%: the count rose through population growth alone. The full decomposition, with the population and rate components in cases for each cancer, is given in Table S4. Where the rate component is large, as it is for breast cancer, the growth in cases is not simply a consequence of there being more Indians of this age. It does not, by itself, show why the rate rose. The 15–49 band aged over the period: the 40–49 share of it rose from 18.9% to 22.3% while the 15–24 share fell from 38.3% to 33.3%. Separating that shift as a third factor leaves the ordering of the four cancers unchanged and the breast finding intact, and it changes the cervical result (Table 1 and Table S7). For breast cancer the rate component is 51.8% rather than 59.0%, with 7.7% attributable to within-band ageing; for colorectal 32.7% with 10.5% ageing; for stomach −47.3% with 24.6% ageing. For cervical cancer, the rate component turns from positive to negative, at −8.1% with 15.5% ageing: once ageing is separated, the whole of the increase in cervical cases is demographic. On within-band standardised rates, the trends are breast +2.36% per year (95% CI 1.90 to 2.83), colorectal +0.76% (0.43 to 1.09), stomach −0.87% (−1.09 to −0.64), and cervical −0.39% (−0.87 to 0.09). Standardisation moves the cervical trend downward, from −0.09% to −0.39% per year. With autocorrelation-robust intervals, the standardised cervical decline does not reach significance. With intervals assuming independent errors, it does (−0.65 to −0.13; Table S7). The segmented fit of the standardised cervical series has the same shape as the crude one, a decline of 1.33% per year to 2012, then a rise interrupted by a decline in 2015–2019 (Table S8). Standardising to India’s 1990 composition instead gives materially the same values (Table S7). Propagated through the GBD uncertainty intervals, the two-factor breast rate component was positive in all 2000 draws. Its share had a 95% interval of 45.4% to 65.9%, which includes values below half. The colorectal rate component was positive in 99% of draws (Table S9). The three-factor decomposition was not simulated, so the uncertainty of its 51.8% breast share is not quantified.

### 3.4. Socio-Demographic Gradient Across the States

**Figure 3:**
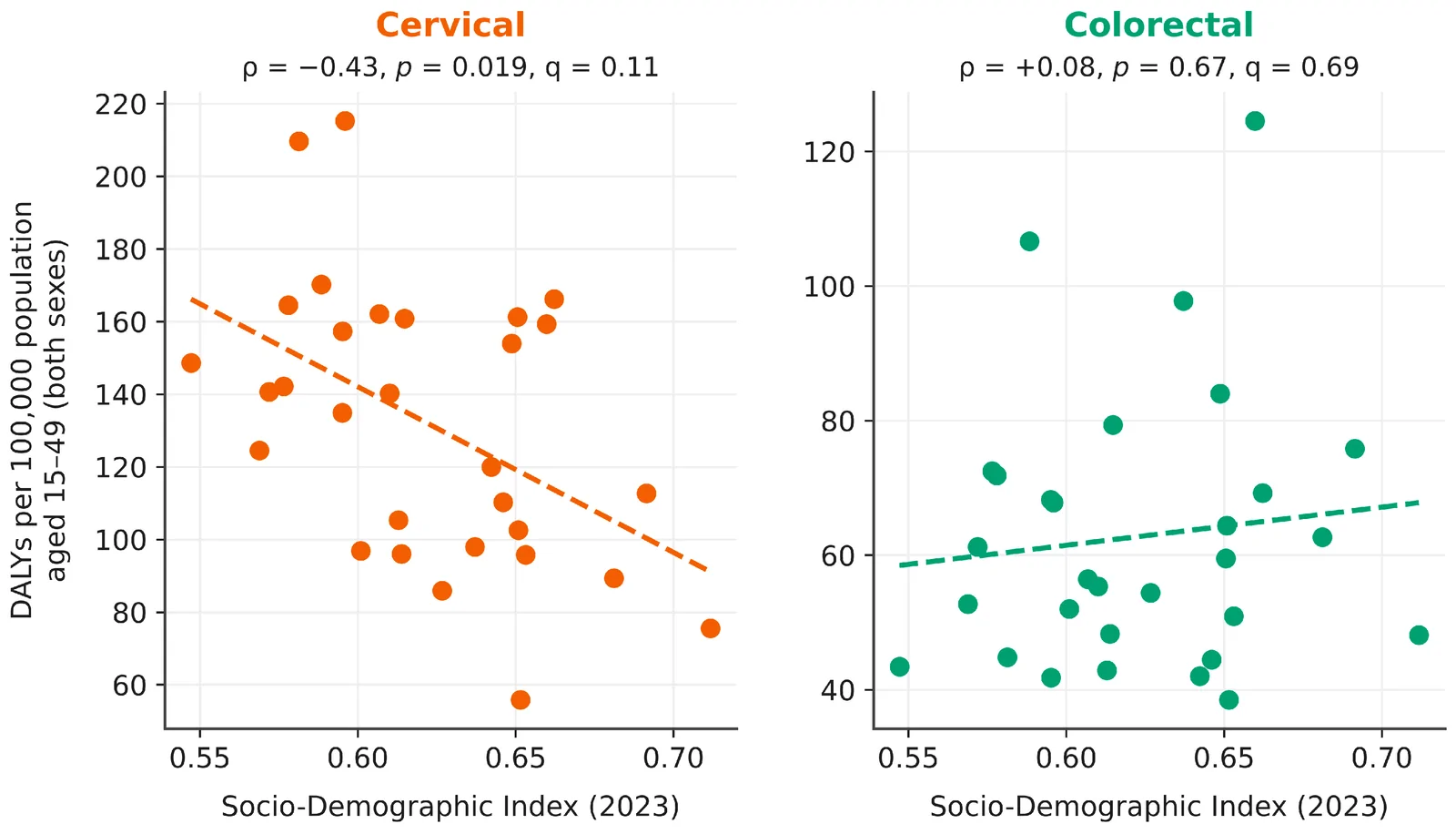
Socio-demographic gradient of early-onset (ages 15–49) cancer DALYs across the 30 states of India, 2023, cervical and colorectal cancer: DALYs per 100,000 population aged 15–49, both sexes, against the 2023 Socio-Demographic Index. Dashed lines are least-squares fits shown for orientation. Above each panel, ρ is the Spearman coefficient, with its unadjusted *p*-value and its Benjamini–Hochberg q-value across the twelve gradient tests. No gradient survives correction, and the gradients are exploratory. Source: GBD 2023 [10].

Point estimates suggested an inverse socio-demographic gradient for cervical cancer that was stronger for mortality and DALYs than for incidence. Across the 30 states, the 2023 cervical DALY rate fell as SDI rose (Figure 3; ρ = −0.43, unadjusted *p* = 0.019; concentration index −0.075), as did the death rate (ρ = −0.44, unadjusted *p* = 0.014). The incidence coefficient was in the same direction but smaller and not nominally significant (ρ = −0.31, *p* = 0.090). None of these associations survived correction for multiple testing: across the twelve gradient tests, the smallest Benjamini–Hochberg q-value is 0.11, for the cervical deaths and DALY gradients. Nor were the gradients statistically distinguishable from one another: the paired bootstrap difference between the DALY and incidence coefficients was −0.11 (95% CI −0.25 to +0.02, *p* = 0.09), and between the deaths and incidence coefficients −0.13 (−0.29 to +0.03, *p* = 0.11). Propagating the GBD uncertainty intervals weakened the pattern further: the cervical DALY coefficient was nominally significant in 50% of 2000 draws, and the incidence coefficient in 18%, and the 95% interval of their difference was −0.48 to +0.23 (Table S9). The pattern is suggestive, not demonstrated. Weighting the states by population gave concentration indices of the same sign and larger magnitude, −0.096 for cervical DALYs (Table S3). The other three gradients did not reach nominal significance across the 30 states: breast, a coefficient close to zero (ρ = −0.11, *p* = 0.56); colorectal, a positive coefficient (ρ = +0.08, *p* = 0.67); and stomach, a negative one (ρ = −0.32, *p* = 0.088), the latter two in the direction cancer-transition theory would predict but underpowered.

### 3.5. State-Level Trends

Breast cancer incidence rose in every one of the 30 states, and in all 30 the 95% CI excluded zero (29 after correction for multiple testing). Five states rose by less than 2% per year: Karnataka (+1.51% per year), Kerala (+1.73%), Madhya Pradesh (+1.92%), Maharashtra (+1.95%), and Tamil Nadu (+1.98%). Three of the five, Karnataka, Kerala and Tamil Nadu, are southern states with population-based cancer registries under the National Cancer Registry Programme [4]. The fastest were spread across the east, northeast, north and west: Jharkhand (+5.22%), Uttarakhand (+4.66%), Odisha (+4.53%), Meghalaya (+4.48%), and Goa (+4.26%). The pace of the rise did not track state SDI (ρ = −0.12, *p* = 0.52). Cervical cancer incidence rose fastest in Arunachal Pradesh (+2.71% per year), Tripura (+2.47%), Delhi (+2.39%), and Meghalaya (+2.36%): three of the four are northeastern states. Its 95% CI lay wholly below zero in five states: Maharashtra (−1.73%), Karnataka (−0.90%), Gujarat (−0.66%), Uttar Pradesh (−0.51%), and West Bengal (−0.41%). The decline is not confined to the west and south: two of the five are large lower-SDI states of the north and east. These statements use each state’s own autocorrelation-robust 95% CI. There are 120 state-by-cancer estimates. Of these, 94 have CIs that exclude zero and 91 remain significant after Benjamini–Hochberg correction across all 120, including every state estimate named above. State-level estimates for all four cancers, with unadjusted and adjusted values, are given in Table S2.

### 3.6. Where the Burden Is Heaviest

**Figure 4:**
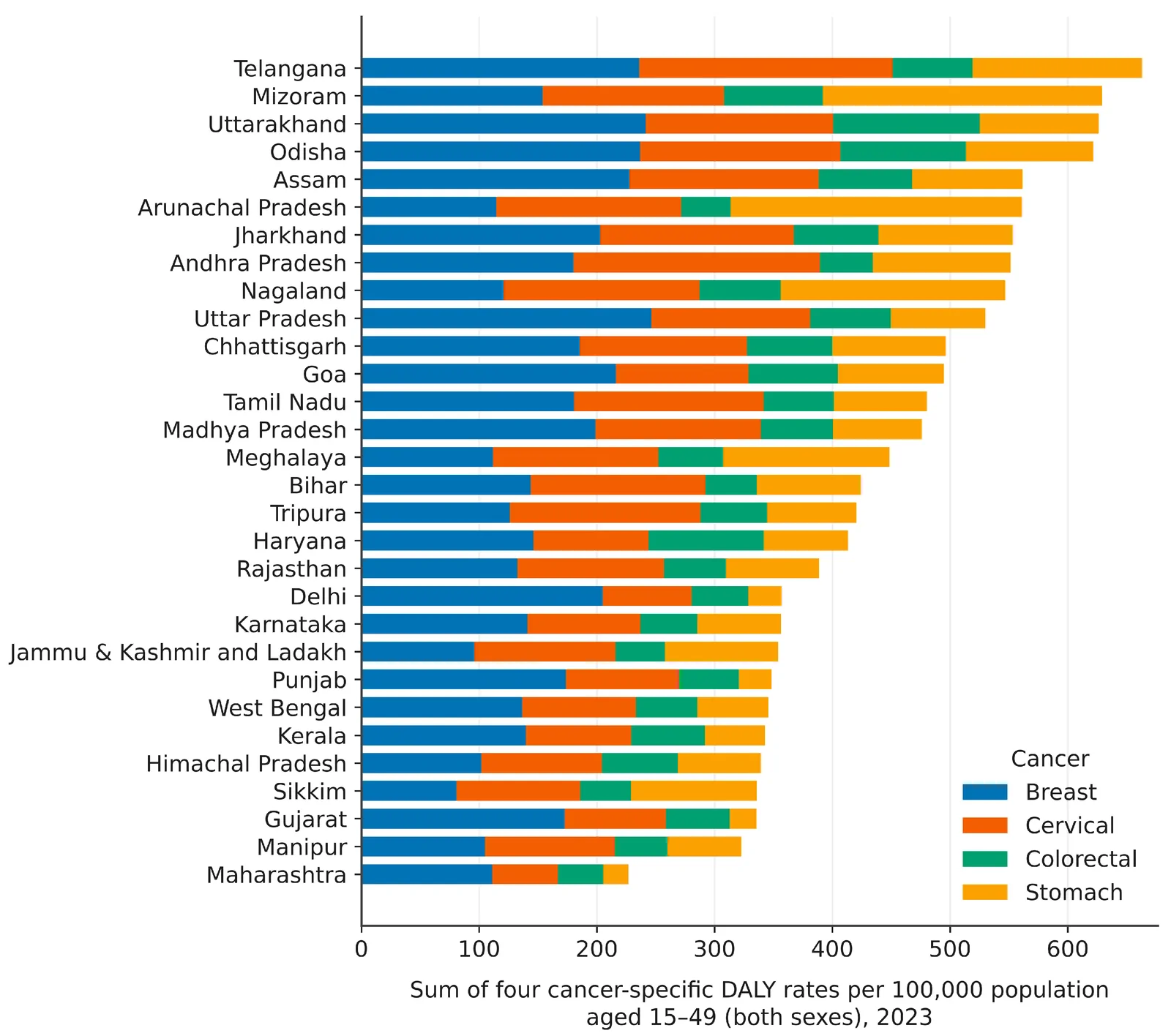
State ranking of early-onset (ages 15–49) cancer burden, 2023: sum of the four cancer-specific DALY rates per 100,000 population aged 15–49, both sexes, showing the contribution of each cancer. The sum is a descriptive measure constructed for this analysis, not a validated index of cancer-control need. Uncertainty in the ranks is given in Table S9. Source: GBD 2023 [10].

Ranking the states by the sum of the four cancer-specific DALY rates in 2023 (Figure 4), the heaviest burden was carried by Telangana (663.2 per 100,000), Mizoram (629.0), Uttarakhand (626.3), Odisha (621.7), and Assam (561.4). The lightest was carried by Maharashtra (226.8), Manipur (322.7), and Gujarat (335.4). The range across the 30 states is 2.9-fold, with a median of 436.4. The ranking is less certain than its point estimates. Across 2000 draws from the GBD uncertainty intervals, the 95% interval of Telangana’s rank was 1st to 10th, and it was in the top five in 82% of draws. Mizoram, Uttarakhand and Odisha were in the top five in 68–71% of draws, and Maharashtra was in the bottom five in all but one draw (Table S9). The states at the top do not reach it by the same route. In Telangana, Uttarakhand, Odisha, and Assam, the largest single contribution is breast cancer. In Mizoram, it is stomach cancer, at 237.1 per 100,000. That figure on its own exceeds the four-cancer total of Maharashtra. The ranking does not sort by SDI. Mizoram and Uttarakhand are in the top third of states by SDI and carry the second and third heaviest burden. Goa, the second highest by SDI, sits in the upper half. Maharashtra and Kerala, of comparable or higher SDI, carry the lightest and the sixth lightest. Bihar, the lowest-SDI state, ranks 16th. What the ranking does show is that the northeastern and eastern states appear repeatedly in the upper half.

Rate and caseload point in different directions. Telangana, first of the 30 states by rate, ranks ninth by caseload. Uttar Pradesh carried 33,695 incident cases in 2023, 19.5% of the national total, but ranks tenth of 30 by rate. Mizoram ranks second by rate and twenty-ninth by count, with 226 cases. Maharashtra carries the lightest rate burden and the sixth largest caseload. Across the 30 states, no monotonic association was detected between the summed DALY rate and the incident case count (ρ = +0.02, *p* = 0.93). The result holds on matched measures: incidence rate against incident count (ρ = −0.04, *p* = 0.84), and DALY rate against DALY count (ρ = +0.05, *p* = 0.80). Across 2000 draws from the GBD uncertainty intervals, the original and the matched incidence correlations never reached nominal significance, and the matched DALY correlation did so once (Table S9). Cancer by cancer, only breast cancer showed a positive association of moderate size (DALY rate against DALY count, ρ = +0.42, *p* = 0.022; Table S5). Five states, Uttar Pradesh, Bihar, Madhya Pradesh, Tamil Nadu and West Bengal, account for 47% of all early-onset cases in these four cancers. The quantities answer different planning questions. The incidence rate describes how common diagnosis is in a state’s population; the DALY rate, the severity of population health loss; the case count, the volume of patients to be diagnosed and treated. None of them measures treatment capacity, which this study does not observe. Both rankings are given in Table S5.

## 4. Discussion

### 4.1. Principal Findings

One national figure for early-onset cancer in India conceals four different stories. Breast cancer is rising steeply, and on the point estimates, more than half of the growth in its cases comes from a rising observed rate rather than from there being more young Indians. Cervical cancer shows no material net national trend over three decades: it fell until 2011 and has risen, unevenly, since. Once within-band ageing is separated, its trend shifts downward, from −0.09% to −0.39% per year, and its rate component is negative. Its health loss appeared to fall more heavily on the lower-SDI states than its incidence did, but that contrast does not survive correction across the twelve gradient tests; the two gradients are not statistically distinguishable, and the pattern is exploratory. Colorectal cancer is rising from a low base. Stomach cancer is falling in rate while its case count rises on population alone. These are four different policy problems in the same age band, and they are not distributed the same way across the country. Nor does the map of rates match the map of cases: across the states, no association between the two was detected.

### 4.2. Breast Cancer: A Rate, Not a Headcount

That 59.0% of the three-decade rise in early-onset breast cancer cases is rate change rather than population growth is the finding with the clearest operational implication. A burden that grows because the population grows can be planned for with capacity. A burden that grows because the observed rate grows is likely to outrun capacity planned on population alone, whatever the reason for the rise. The rise was steepest in states as different as Jharkhand and Uttarakhand, each above +4.5% per year. It was slowest in Karnataka, Kerala, Madhya Pradesh, Maharashtra and Tamil Nadu, all below +2% per year. That group spans much of the range of state SDI. Across all states, the pace of the rise did not track it. Subnational registry-based estimates place India’s heaviest female breast cancer health loss in Tamil Nadu, Telangana, Karnataka and Delhi, above the eastern and north-eastern states [41]. None of the six states where it is now climbing fastest (Jharkhand, Uttarakhand, Odisha, Meghalaya, Goa and Arunachal Pradesh) is among them. That suggests the map is shifting. A GBD 2023 comparison of female breast cancer in China, India and the United States decomposed India’s change into population growth, population ageing and age-specific rate change. It likewise attributed the rise to demographic and adverse epidemiological factors together [42].

Two competing explanations deserve weight, and the design cannot separate them. The first is ascertainment. India’s cancer registry programme has expanded since 1982. Population-based registries still cover about a tenth of the population [4]. Part of a measured rise in incidence might therefore reflect the detection of disease that would previously have gone unrecorded, rather than an increase in disease itself. GBD cancer estimation is anchored on mortality and on mortality-to-incidence ratios, and adjusts for data quality [31]. It cannot fully separate the two. This is the most important alternative explanation for the breast cancer finding. The second is that the rise is real. It could reflect the reproductive and metabolic transition that accompanies development: later childbearing, lower parity, and central obesity [1]. The two are not mutually exclusive, and the acceleration of the series after 2012 is consistent with either. What the decomposition establishes is narrower and still useful. On whichever mechanism, the growth in cases is not only a function of there being more Indians of this age. That conclusion survives the removal of within-band ageing, which accounts for 7.7% of the rise against a rate component of 51.8%. In the two-factor decomposition, it also survives the GBD uncertainty intervals, across which the rate component stayed positive. The size of the rate share is less certain: its 95% interval in the two-factor simulation runs from 45.4% to 65.9%, and the three-factor share was not simulated.

### 4.3. Cervical Cancer: A Reversed Decline and a Suggestive Gradient

Three things should be kept apart here: what this study shows, what earlier work suggests, and what remains to be tested. What this study shows is narrow. Nationally, early-onset cervical incidence fell for two decades and has risen, unevenly, since, with no net change on the crude band rate and a downward shift once within-band ageing is removed. Across the states, the point estimates of the cervical death and DALY gradients were more strongly inverse than the incidence gradient, which ran in the same direction. None survives correction for multiple testing; the gradients are not distinguishable from one another, and the incidence coefficient of −0.31 lies within the range that 30 states cannot resolve. A non-significant incidence gradient is not evidence of uniform incidence. What earlier work suggests is a mechanism. Where mortality is more unequal than incidence, screening, stage at presentation and access to treatment are the usual explanations. Population-based registry evidence from Kerala shows that women with less education present at more advanced stages and survive less well. The survival gradient was significant for breast cancer and in the same direction for cervical cancer [43]. Nationally, age-standardised cancer mortality among women aged 30–69 is more than twice as high in the illiterate as in the most educated [25]. What remains to be tested is whether any of these factors explain interstate differences in early-onset cervical outcomes. Screening, stage, treatment access, and financial protection were not measured here, and the SDI is a broad composite that cannot identify which component of development is at work. There is a case for revisiting the question at the district level, where the number of units would support the comparison, and with measured screening and stage data.

India has both an HPV vaccine and a screening programme, and the efficacy evidence from Indian cohorts is strong [15]. The difficulty seems to have been in delivery and coverage [16]. Only 1.2% of women aged 15–49 have ever been screened [28]. Registry data from the same period point the same way. Breast cancer incidence rose significantly in almost every population-based registry with trend data, while cervical cancer incidence fell significantly in ten of them [4]. National GBD analyses have documented the long decline of cervical cancer in India and its persisting state-level heterogeneity [27]. International comparisons report cervical cancer incidence rising among adults aged 15–49 globally over this period, fastest in the high-SDI region [44]. Over the full period, India does not share that pattern, though its early-onset series has turned upward since 2011. That reversal is the part of the cervical trajectory most in need of confirmation from registry series, since changes in the input data could produce it. An inverse gradient of the same shape has been reported for childhood cancer on GBD 2023, where age-standardised years of life lost in 2023 fell as country-level Socio-Demographic Index rose [45]. A subnational GBD analysis of central nervous system cancer across the provinces of China reports the divergence directly: age-standardised incidence correlated positively with the Socio-Demographic Index while deaths and disability-adjusted life years correlated negatively [46]. Our point estimates are consistent with both. Three of the four fastest rises in early-onset cervical incidence are in the northeastern states of Arunachal Pradesh, Tripura and Meghalaya, where registry data would be most informative.

Whether this early-onset pattern differs from the all-age one is a natural question to ask of it. The GBD subnational analysis of all-age cancer in India documented wide interstate variation in cancer burden and in cancer-specific composition, and the same divergence in direction: between 1990 and 2016 the age-standardised incidence of breast cancer rose by 40.7% while cervical and stomach cancer each fell by 39.7% [11]. A formal comparison of the two age windows was not undertaken here and would be a useful extension; the claims made in this paper are confined to adults aged 15–49.

### 4.4. Colorectal and Stomach Cancer: A Transition in Progress

Colorectal cancer is rising and stomach cancer is falling. That is the pattern expected of an epidemiological transition in progress. In global colorectal analyses on GBD data, population growth is likewise the primary factor behind the rising absolute burden [47], as it is here. A GBD 2023 decomposition of all-age gastric cancer in India separated the demographic component into population growth and population ageing, and found the ageing component the larger of the two (87.8% against 58.3% of the change in cases) [48]. That analysis puts India’s all-age age-standardised gastric incidence trend at −0.91% per year. Our within-band standardised early-onset estimate of −0.87% (−1.09 to −0.64) is close to it. The crude band rate of −0.52% is not. The colorectal gradient had a positive coefficient and the stomach gradient a negative one, in the direction cancer-transition theory would predict. Neither reached significance across 30 states. Early-onset colorectal cancer is rising in country after country, and its risk factors seem to be the metabolic and dietary ones that accompany development [6,7,17]. India’s rates remain low by international standards [6]. That is precisely why the trend matters. A low rate rising at 1.11% per year in a population of this size could produce large absolute numbers within a generation, and the segmented fit suggests the rise has steepened since 2018.

### 4.5. Implications for Prioritisation and Further Assessment

The findings support priorities for further assessment rather than direct prescriptions. This study does not measure vaccination or screening coverage, stage at diagnosis, treatment access, health-system capacity, or cost-effectiveness, and these decide where an additional resource would do most good. Within that limit, three distinctions follow from the analysis. States with high rates warrant investigation of prevention and early-detection gaps: the northeastern and eastern states recur in the upper half of the ranking, and cervical incidence is rising fastest in several northeastern states. States with large caseloads, Uttar Pradesh first among them, require greater absolute diagnostic and treatment capacity whatever their rate. Breast cancer, whose observed rate is rising in every state, warrants assessment of early-detection capacity where the rate is climbing fastest and not only where the count is currently highest. Across all of these, the four cancers are better monitored separately at the state level than as a single cancer aggregate.

The burden map also generates a hypothesis about financial protection. Catastrophic expenditure varied eight-fold across states in 2014 [21]. Publicly funded insurance in India does not cover outpatient care [23,24], and cancer carries the highest odds of distress financing of any illness group [19]. If the states with a high early-onset burden are also the states with weak financial protection, the household cost of an early-onset cancer compounds. This analysis measures burden and not expenditure. The overlap is a hypothesis for testing, not a result of this study. Ecological analyses of this kind have been used elsewhere to frame such hypotheses [49], and are routine in GBD subnational work [46].

### 4.6. Strengths and Limitations

The strengths of this analysis are its coverage and its reproducibility. Four cancers are characterised for every state across 34 years on a single consistent set of estimates, replacing a national figure with a state-level map for each cancer. Three independent methods are applied to the same extract, and they answer different questions: the trend analysis asks what is changing, the gradient analysis asks who bears it, and the decomposition asks whether the change is a matter of rates or of numbers. The trend intervals allow for autocorrelation, the shape of each national trend is described by segmented fits, and the main cross-sectional results were tested against the GBD uncertainty intervals. The analysis-ready extract, the five-year age band extract, the unrounded state Socio-Demographic Index values, and the complete analysis script are supplied, so that every reported number, table and figure can be regenerated independently.

The limitations are those of the data and of the design. A rise in measured incidence across three decades may in part reflect improving registry coverage and diagnostic capacity rather than a rise in underlying risk. No design based on these estimates can separate the two. The estimates are modelled rather than based on primary registry data. India’s population-based cancer registries cover only part of the country and unevenly across states [4]. Estimates for data-sparse states therefore rely more on statistical modelling and covariates than on observed registry data. Uncertainty is correspondingly wider for the small northeastern states and for the earliest years. GBD posterior draws were not available, so the uncertainty intervals could be propagated only approximately, by independent draws that ignore the correlation of estimates across states and years. On that approximation, the state ranks are less certain than their point estimates, the cervical gradients are less stable, and the absence of a rate–caseload association holds (Table S9). The trend CIs reflect the fitted trend conditional on the GBD point estimates, not the full uncertainty of the underlying estimates. The EAPC is an average over 34 years. Most of the national series are not log-linear. The segmented fits describe their shape, but their intervals are conditional on the selected joinpoints, and the state series were not segmented. The design is ecological, so no causal or individual-level inference is available from it, and the SDI analysis is exploratory. With 30 states, the gradient analyses are underpowered. Deaths and DALYs at ages 15–49 do not capture deaths at older ages among patients diagnosed before 50. Lung cancer, which causes more cancer deaths in India than any other site, was not studied.

The main-text decomposition is two-factor, and its rate component therefore carries any shift in age composition within the 15–49 band. That shift is not negligible. Within India’s 15–49 population, the colorectal rate has fallen at ages 15–29 while rising, though not significantly, at 40–44 [29]. It was tested directly. The three-factor decomposition and the within-band standardised trends reported above and in Table S7 leave the direction of every trend and the ordering of the four cancers intact, and change one result: cervical incidence, with no net trend on the crude band rate, shifts downward once composition is removed, and its rate component turns negative. This test was possible only at the national level. The state-level trends in Table S2 and the twelve gradient tests remain on crude band rates, and states differ in their within-band composition. Differences in within-band age composition may therefore influence the interstate gradients. Their direction and magnitude require state-level age-standardised analysis. The residual union-territory aggregate is excluded. With 30 states, the analysis could not have detected a gradient difference of the size observed. The smallest coefficient detectable at 80% power is |ρ| = 0.49, and the observed cervical mortality gradient of −0.44 was estimated with about 70% power. The nulls reported here are therefore a limit of the design and not evidence of absence. Finally, the financial-protection argument in this paper is drawn from the published literature and not measured here. Linking state-level burden to state-level out-of-pocket and catastrophic expenditure directly is the next step, and the data to do it exist.

## 5. Conclusions

The early-onset cancer burden in India is not uniform, and the national figure conceals a pattern that differs by cancer and by state. Breast cancer cases are growing faster than the population, through a rising observed rate, and the rate is rising fastest outside the southern states where the burden has historically been measured. Whether that reflects more disease, more detection, or both cannot be settled from these estimates. Cervical cancer fell for two decades and has risen, unevenly, since, with little net change over the period. Once within-band ageing is removed, its trend shifts downward and its rate component turns negative. Its health loss appeared concentrated in lower-SDI states, but that pattern is exploratory and needs confirmation. Colorectal cancer is rising from a low base and stomach cancer is falling on rate: a transition in progress. The states with the highest rates are not the states with the most cases. Rates point to where prevention and detection gaps should be investigated. Caseload points to where service volume is greatest. Both are needed, and neither alone tells a state where to act.

## Figures and Tables

**Table 1:** Early-onset (ages 15–49) breast, cervical, colorectal, and stomach cancer in India, 1990–2023: burden, trend, decomposition, and socio-demographic gradient.

Indicator	Breast	Cervical	Colorectal	Stomach
Incidence per 100,000, 2023 (95% UI)	9.63 (7.19–13.26)	8.18 (5.28–12.48)	1.92 (1.49–2.42)	1.91 (1.33–2.55)
Incidence per 100,000 women, 2023 (95% UI)	19.63 (14.69–27.19)	16.87 (10.88–25.72)	—	—
Deaths per 100,000, 2023 (95% UI)	3.46 (2.55–4.56)	2.50 (1.67–3.69)	1.18 (0.91–1.49)	1.51 (1.06–2.02)
DALYs per 100,000, 2023 (95% UI)	178.2 (132.1–233.9)	127.5 (84.5–187.5)	59.5 (46.1–75.0)	74.4 (52.4–99.2)
EAPC in incidence, % per year (95% CI)	+2.72 (2.19, 3.25)	−0.09 (−0.61, 0.43)	+1.11 (0.75, 1.48)	−0.52 (−0.75, −0.28)
EAPC in incidence, within-band standardised, % per year (95% CI)	+2.36 (1.90, 2.83)	−0.39 (−0.87, 0.09)	+0.76 (0.43, 1.09)	−0.87 (−1.09, −0.64)
Incident cases, 1990	15,550	32,715	5040	8996
Incident cases, 2023	76,806	65,273	15,344	15,250
Change in cases, %	+394	+100	+204	+70
Rate-change share of the rise, two-factor, %	59.0	7.5	42.9	−22.2
Rate-change share of the rise, three-factor, %	51.8	−8.1	32.7	−47.3
SDI gradient in DALYs, Spearman ρ (*p*; q)	−0.11 (0.56; 0.69)	−0.43 (0.019; 0.11)	+0.08 (0.67; 0.69)	−0.32 (0.088; 0.26)

## Data Availability

All data analysed in this study are publicly available. Cancer burden estimates were obtained from the GBD Results tool (https://vizhub.healthdata.org/gbd-results/, accessed on 15 July 2026) and the Socio-Demographic Index from the Global Health Data Exchange (https://ghdx.healthdata.org/, accessed on 5 July 2026), both maintained by the Institute for Health Metrics and Evaluation, University of Washington, Seattle, WA, USA. The analysis-ready data extract, the five-year age band extract, the unrounded state Socio-Demographic Index values and Tables S1–S9 are provided as File S1; the complete analysis script is provided as File S2. The script reads the data workbook directly and regenerates Tables S1–S9, every cell of Table 1, and Figure 1, Figure 2, Figure 3 and Figure 4.

## Supplementary Materials

The following supporting information can be downloaded at: https://www.mdpi.com/article/10.3390/cancers18213580/s1, File S1: Supplementary data workbook, containing the analysis-ready GBD 2023 extract, the five-year age band extract, the unrounded state Socio-Demographic Index values for 2023, and Tables S1–S9; File S2: Analysis script (Python 3.12 source, supplied as a .txt file, to be renamed .py to run); File S3: Completed GATHER checklist. Table S1: National estimated annual percentage change (EAPC) in early-onset incidence, deaths and DALYs, 1990–2023, with autocorrelation-robust and ordinary confidence intervals and trend diagnostics; Table S2: State-level EAPC in early-onset incidence, 1990–2023, with Benjamini–Hochberg q-values; Table S3: Socio-demographic (SDI) gradient across states, 2023, with equally weighted and population-weighted concentration indices; Table S4: Decomposition of the change in incident cases, 1990–2023; Table S5: Composite state burden ranking (four-cancer DALY rate) and rate–caseload correlations on original and matched measures, 2023; Table S6: Sex-specific denominator: sensitivity analysis and female-specific rates; Table S7: Sensitivity of the national trend and decomposition results to within-band age composition; Table S8: Segmented (joinpoint-type) trends and average annual percentage change, India, 1990–2023; Table S9: Approximate propagation of GBD uncertainty intervals into the state ranking, gradients, rate–caseload correlations and two-factor decomposition.

## Abbreviations

AAPC	average annual percentage change
CI	confidence interval
DALY	disability-adjusted life year
EAPC	estimated annual percentage change
GATHER	Guidelines for Accurate and Transparent Health Estimates Reporting
GBD	Global Burden of Disease
HPV	human papillomavirus
IHME	Institute for Health Metrics and Evaluation
SDI	Socio-Demographic Index
STROBE	Strengthening the Reporting of Observational Studies in Epidemiology
UI	uncertainty interval
WHO	World Health Organization
