# Supplementary material for: Early-Onset Breast, Cervical, Colorectal and Stomach Cancer Across the States of India, 1990–2023: Divergent Trends and a Mismatch Between Burden Rate and Caseload
Source: Cancers (Basel). 2026 Nov 7;18(21):3580. doi: 10.3390/cancers18213580 (PMC13761526; doi:10.3390/cancers18213580)
Supplement: Supplementary file 1 [file cancers-18-03580-s001.zip › File_S3_GATHER_checklist.pdf]

# File S3: GATHER checklist

*Early-onset breast, cervical, colorectal and stomach cancer across the states of India, 1990–2023: divergent trends and a mismatch between burden rate and caseload*

Reporting follows the Guidelines for Accurate and Transparent Health Estimates Reporting (GATHER), a checklist of 18 items. Item numbers and item text below are those of the published checklist: Stevens GA, Alkema L, Black RE, Boerma JT, Collins GS, Ezzati M, et al. Guidelines for Accurate and Transparent Health Estimates Reporting: the GATHER statement. Lancet 2016;388(10062):e19–e23, Table 1. GATHER is published under a CC BY licence and is reproduced here with citation. Section headings and the two data-input sub-groups follow the published structure. Cross-references are to the section headings of the revised manuscript.

| Item | GATHER checklist item | Where reported in this submission |
|---|---|---|
| **Objectives and funding** | | |
| 1 | Define the indicator(s), populations (including age, sex, and geographic entities), and time period(s) for which estimates were made. | Abstract (Background/Objectives; Methods). Section 1, Introduction, final paragraph. Sections 2.2, Case Definition and Variables, and 2.3, Locations. Indicators: age-specific incidence, mortality and DALYs per 100,000. Population: adults aged 15–49 years, both sexes combined and by sex. Geographic entities: India and its GBD subnational units; the 30 named states and union territories in the state-level ecological analyses. Period: 1990–2023. |
| 2 | List the funding sources for the work. | Funding: this research received no external funding. |
| **Data inputs** | | |
| *For all data inputs from multiple sources that are synthesized as part of the study:* | | |
| 3 | Describe how the data were identified and how the data were accessed. | Section 2.1, Study Design and Data Source. Cancer burden estimates were extracted from the GBD Results tool (https://vizhub.healthdata.org/gbd-results/); the Socio-Demographic Index was obtained from the Global Health Data Exchange (https://ghdx.healthdata.org/). Both are open access. The burden estimates were extracted on 15 July 2026, from the GBD 2023 release of 12 October 2025, and the SDI file (IHME_GBD_2023_SDI_1950_2023_Y2025M10D12) was accessed on 5 July 2026; the five-year age band extract used in the sensitivity analysis was taken from the same release on 6 September 2026; the extraction date is stated in Methods and in the README sheet of File S1, which also records the full query specification (measures, metrics, causes, sexes, ages, years, locations). |
| 4 | Specify the inclusion and exclusion criteria. Identify all ad-hoc exclusions. | Sections 2.2, Case Definition and Variables, and 2.3, Locations. Included: four causes (breast, cervical, colon and rectum, stomach cancer); ages 15–49; both sexes and each sex; incidence, deaths and DALYs; number and rate metrics; 1990–2023. Ad-hoc exclusions, both declared: the residual “Other Union Territories” aggregate and the national “India” location are excluded from the state-level analyses (state trends, SDI gradient and burden ranking), leaving 30 |

| | | |
|---|---|---|
| | | units; no urban or rural splits were used. No other exclusions were applied and no estimates were dropped. |
| 5 | Provide information on all included data sources and their main characteristics. For each data source used, report reference information or contact name/institution, population represented, data collection method, year(s) of data collection, sex and age range, diagnostic criteria or measurement method, and sample size, as relevant. | Not applicable in the sense intended by GATHER: this study synthesised no primary data sources. Its sole health input is a published set of modelled estimates (GBD 2023), reported under item 7. The primary inputs underlying GBD 2023 for India — population-based cancer registries, the Sample Registration System, vital registration and survey data — are documented by IHME in the GBD 2023 sources catalogue on the GHDx; IHME, University of Washington, is the custodian. Their coverage limitations are discussed under item 6. |
| 6 | Identify and describe any categories of input data that have potentially important biases (e.g., based on characteristics listed in item 5). | Section 4.6, Strengths and Limitations. Two biases are identified. First, India's population-based cancer registries cover only part of the country and unevenly across states, so estimates for data-sparse states rely more on statistical modelling and covariates than on observed registry data, and uncertainty is wider for the small northeastern states and for the earliest years. Second, ascertainment: a rise in measured incidence across three decades may in part reflect improving registry coverage and diagnostic capacity rather than a rise in underlying risk. The latter is also discussed as the principal competing explanation for the breast cancer finding in Section 4.2, Breast Cancer: A Rate, Not a Headcount. |
| *For data inputs that contribute to the analysis but were not synthesized as part of the study:* | | |
| 7 | Describe and give sources for any other data inputs. | Section 2.1, Study Design and Data Source. Two inputs, both used without modification: (i) GBD 2023 burden estimates (incidence, deaths, DALYs; counts, rates and 95% uncertainty intervals) for India and its subnational units, from the GBD Results tool; (ii) the GBD 2023 Socio-Demographic Index for 2023, from the GHDx, matched to the burden estimates by GBD location name, with the GBD location identifiers listed in File S1. The GBD Results tool is reference 10, and the SDI release is identified by its file name in File S1; the GBD 2023 estimation methods are references 30 and 31. |
| *For all data inputs:* | | |
| 8 | Provide all data inputs in a file format from which data can be efficiently extracted (e.g., a spreadsheet rather than a PDF), including all relevant meta-data listed in item 5. For any data inputs that cannot be shared because of ethical or legal reasons, such as third-party ownership, provide a contact name or the name of the institution that retains the | File S1 (Excel workbook). The sheet "Analysis-ready extract" contains the complete tidy extract underlying every result except the within-band standardised analyses: 71,808 rows, with the value and the upper and lower 95% uncertainty bounds for each estimate. The sheet "Five-year extract" (5,236 rows; India by seven five-year age |

| | | |
|---|---|---|
| | right to the data. | groups) underlies the within-band standardised analyses (Tables S7 and S8). The sheet "SDI (2023)" contains the unrounded state SDI values, with GBD location identifiers, on which every SDI statistic is computed. The README sheet records the data source, query specification, extraction date and methods notes. Nothing is withheld. The primary data underlying the GBD estimates are held by the Institute for Health Metrics and Evaluation, University of Washington, Seattle, named as custodian in the Data Availability Statement. |
| **Data analysis** | | |
| 9 | Provide a conceptual overview of the data analysis method. A diagram may be helpful. | Section 2.4, Statistical Analysis. Three primary analyses are applied to the same extract: (i) a trend analysis summarising each rate series by its estimated annual percentage change, with Newey–West autocorrelation-consistent confidence intervals; (ii) a cross-sectional ecological analysis relating 2023 state burden to 2023 state SDI; (iii) a two-factor decomposition separating the 1990–2023 change in case counts into population growth and rate change. A subordinate analysis compares two of the gradients from (ii) to each other by paired bootstrap, and the state ranking by burden rate is compared with the ranking by caseload. A national sensitivity analysis repeats (i) and (iii) on seven five-year age bands (Table S7). Three further analyses were added in revision at the reviewers' request: segmented (joinpoint-type) fits of the national series (Table S8), the rate–caseload comparison on matched measures (Table S5), and an approximate propagation of the GBD uncertainty intervals (Table S9). No diagram is included; the analyses are independent and are not chained. |
| 10 | Provide a detailed description of all steps of the analysis, including mathematical formulae. This description should cover, as relevant, data cleaning, data pre-processing, data adjustments and weighting of data sources, and mathematical or statistical model(s). | Section 2.4, Statistical Analysis, gives the formulae for the EAPC ($\ln(\text{rate}) = \alpha + \beta \cdot \text{year} + \varepsilon$; $\text{EAPC} = 100 \times (\exp(\beta) - 1)$, with a 95% CI from Newey–West heteroskedasticity- and autocorrelation-consistent standard errors, bandwidth three years, small-sample correction, t distribution with 32 degrees of freedom) and the health concentration index ($C = 2 \cdot \text{cov}(y, R)/\mu$, with states equally weighted and, alternatively, population-weighted). It also describes the segmented log-linear model (up to three joinpoints, number chosen by the Bayesian information criterion), the Das Gupta–type two-factor decomposition, the paired bootstrap comparison of two gradients, the Benjamini–Hochberg |

| | | |
|---|---|---|
| | | correction, the log-normal approximation used to propagate the GBD uncertainty intervals, and, for the sensitivity analysis, within-band direct standardisation to the WHO World Standard Population and the Das Gupta symmetric three-factor decomposition into population size, age structure and age-specific rates. Methods are attributed to their sources where one exists (references 34–40). The exact computations, including the decomposition formulae, the standard-population weights and the log-normal standard deviation (sd = (ln upper UI − ln lower UI)/3.92), are implemented and documented in File S2, which reads File S1 directly. No data cleaning, adjustment or weighting was applied: the extract is used exactly as downloaded, with no missing values. |
| 11 | Describe how candidate models were evaluated and how the final model(s) were selected. | Not applicable in the sense intended by GATHER: no new estimation model of disease burden was fitted. The primary estimators were specified before analysis: a log-linear trend on year, Spearman rank correlation with the concentration index, a Das Gupta–type decomposition, and a paired bootstrap. In revision, the trend analysis was supplemented by segmented log-linear models in which the number of joinpoints (zero to three) was chosen by the Bayesian information criterion (Table S8), and Newey–West intervals at alternative bandwidths are reported alongside the primary intervals (Table S1). |
| 12 | Provide the results of an evaluation of model performance, if done, as well as the results of any relevant sensitivity analysis. | No model-performance evaluation was done, because no estimation model of disease burden was fitted (item 11). Goodness of fit of the trend models is reported in Table S1 ($R^2$, residual autocorrelation, Durbin–Watson statistic, and a test of an added quadratic term with Newey–West standard errors) and Table S8. Sensitivity and robustness analyses are reported in full. (i) Table S6 repeats the headline analyses for breast and cervical cancer on a female-only denominator; every conclusion is unchanged (breast EAPC +2.67%/yr vs +2.72%/yr; cervical −0.12%/yr vs −0.09%/yr; cervical DALY–SDI gradient $\rho = -0.44$, $p = 0.015$ vs $\rho = -0.43$, $p = 0.019$; breast rate share 58.3% vs 59.0%). (ii) Table S7 repeats the national trend and decomposition on seven five-year age bands: every direction and ordering is unchanged, the breast rate component is 51.8% against 59.0%, and cervical incidence, with no net trend on the crude band rate, shifts downward once composition is removed (−0.39%/yr) and |

| | | |
|---|---|---|
| | | its rate component turns negative. (iii) Section 3.4 reports a paired bootstrap testing whether the cervical mortality gradient differs from the cervical incidence gradient; it does not at 30 states. (iv) Table S1 reports trend intervals under alternative error assumptions, and Table S8 the segmented fits. (v) Table S5 repeats the rate–caseload comparison on matched measures. (vi) Table S9 propagates the GBD uncertainty intervals approximately into the ranking, cervical SDI gradients, rate–caseload correlations and two-factor decomposition; the three-factor decomposition was not simulated. All are regenerated by File S2. |
| 13 | Describe methods for calculating uncertainty of the estimates. State which sources of uncertainty were, and were not, accounted for in the uncertainty analysis. | Sections 2.2, Case Definition and Variables, and 2.4, Statistical Analysis. Accounted for: the uncertainty of each fitted trend, as a 95% CI from Newey–West autocorrelation-consistent standard errors (with ordinary intervals for comparison); the sampling uncertainty of each correlation, as a two-sided p value with Benjamini–Hochberg adjustment; and the uncertainty of the difference between two gradients, as a bootstrap percentile interval. The uncertainty of the GBD estimates themselves is accounted for only approximately: GBD posterior draws are not distributed through the public Results tool, so each 2023 estimate, and each 1990 and 2023 national estimate used in the two-factor decomposition, was represented by a log-normal distribution derived from its 95% UI and 2000 independent draws were used to recompute the ranking, cervical SDI gradients, rate–caseload correlations and two-factor decomposition (Table S9). Not accounted for: the uncertainty of the GBD estimates in the trend intervals, which are conditional on the GBD point estimates, and in the three-factor decomposition; and the correlation of GBD estimates across states and years. The GBD 95% uncertainty intervals are retained for every estimate in the extract and displayed as bands in Figure 1. These limitations are stated in Section 4.6, Strengths and Limitations. |
| 14 | State how analytic or statistical source code used to generate estimates can be accessed. | File S2 (analysis script, Python 3.12) is submitted with the manuscript and is intended for publication alongside it. It reads File S1 and regenerates Tables S1–S9, the bootstrap comparison, every cell of Table 1, and Figures 1–4. Named in the Data Availability Statement. The file is supplied as plain text (.txt) so that it remains machine-readable; rename it to .py to run. |

| **Results and discussion** | | |
|---|---|---|
| 15 | Provide published estimates in a file format from which data can be efficiently extracted. | File S1 (Excel workbook): the full analysis-ready extract, the five-year age band extract for India, the unrounded state Socio-Demographic Index values and Tables S1–S9, containing every reported EAPC and confidence interval, every SDI gradient coefficient and p value, every concentration index, the decomposition results and the composite state ranking. Table 1 in the main text is a Word table object, not an image. |
| 16 | Report a quantitative measure of the uncertainty of the estimates (e.g., uncertainty intervals). | 95% confidence intervals (Newey–West) are reported for all four national EAPCs in Section 3.2, Divergent National Trends, and in Table 1, for the national trends in deaths and DALYs in Table S1, for all 120 state-level EAPCs in Table S2, and for the segmented trends in Table S8. Two-sided p values are reported for all correlations in Table S3, with Benjamini–Hochberg q values for the twelve gradient tests, for the cervical gradients in Section 3.4, and in Table 1; the Abstract reports the coefficients. The bootstrap comparison is reported with a 95% percentile interval and a p value. GBD 95% uncertainty intervals are reported for the 2023 national rates in Section 3.1 and Table 1, retained for every estimate in the extract, shown as bands in Figure 1, and propagated approximately in Table S9. |
| 17 | Interpret results in light of existing evidence. If updating a previous set of estimates, describe the reasons for changes in estimates. | Section 4.1, Principal Findings and every subsequent subsection, which set each finding against prior GBD analyses, registry-based Indian estimates, and subnational analyses from India and elsewhere (references 1, 4, 6–7, 11, 15–17, 19, 21, 23–25, 27–29, 31, 41–49). Section 4.3, Cervical Cancer: A Reversed Decline and a Suggestive Gradient, also states explicitly that a formal comparison with the all-age pattern was not undertaken here. This study does not update or supersede a previous set of estimates: it is a secondary analysis of the published GBD 2023 estimates, which are reproduced unchanged, so no reconciliation of differing estimates is required. |
| 18 | Discuss limitations of the estimates. Include a discussion of any modelling assumptions or data limitations that affect interpretation of the estimates. | Section 4.6, Strengths and Limitations. Covers: ascertainment, in that a measured rise in incidence may in part reflect improving registry coverage and diagnostic capacity; estimates being modelled rather than primary registry data; uneven registry coverage across states; the approximate propagation of GBD uncertainty and the conditional nature of the trend intervals (item 13); the EAPC as an average over a period in |

| | | |
|---|---|---|
| | | which most national series are not log-linear, with segmented fits conditional on the selected joinpoints; the ecological design, which precludes causal and individual-level inference, and the exploratory status of the SDI analysis; limited power at 30 states (Section 4.6), with no gradient surviving correction for multiple testing and the cervical incidence and mortality gradients not statistically distinguishable from one another (Sections 3.4, 4.1 and 4.3); deaths and DALYs at ages 15–49 not capturing later deaths of patients diagnosed before 50; the two-factor main-text decomposition, whose rate component carries within-band age-structure shifts, tested directly at national level in Table S7 but not for the state-level trends or gradients, where the direction and magnitude of its effect would require state-level age-standardised analysis; exclusion of the residual union-territory aggregate and of lung cancer; and the financial-protection argument being drawn from the literature rather than measured here. |

**Note.** *This study analysed only publicly available, aggregated GBD 2023 estimates. No primary data were collected, no new estimation model was fitted, and the GBD estimates are reproduced unchanged. Items 5 and 11 are therefore reported as not applicable in the sense intended by GATHER, and item 12 is inapplicable only in respect of model performance — the sensitivity and robustness analyses it also asks for were performed and are reported in full. In every case the reason is stated rather than the item left blank. All 18 items are addressed.*
